# Selective blockade of spinal D2DR by *levo*-corydalmine attenuates morphine tolerance via suppressing PI3K/Akt-MAPK signaling in a MOR-dependent manner

**DOI:** 10.1038/s12276-018-0175-1

**Published:** 2018-11-14

**Authors:** Wen-Ling Dai, Xin-Tong Liu, Yi-Ni Bao, Bing Yan, Nan Jiang, Bo-Yang Yu, Ji-Hua Liu

**Affiliations:** 1 Jiangsu Key Laboratory of TCM Evaluation and Translational Research, School of Traditional Chinese Pharmacy, China Pharmaceutical University, Nanjing, Jiangsu 211198 China; 2 School of Life Science and Technology, China Pharmaceutical University, Nanjing, Jiangsu 211198 China; 3 State Key Laboratory of Natural Medicines, China Pharmaceutical University, Nanjing, Jiangsu 210009 China

## Abstract

Morphine tolerance remains a challenge in the management of chronic pain in the clinic. As shown in our previous study, the dopamine D2 receptor (D2DR) expressed in spinal cord neurons might be involved in morphine tolerance, but the underlying mechanisms remain to be elucidated. In the present study, selective spinal D2DR blockade attenuated morphine tolerance in mice by inhibiting phosphatidylinositol 3 kinase (PI3K)/serine–threonine kinase (Akt)-mitogen activated protein kinase (MAPK) signaling in a μ opioid receptor (MOR)-dependent manner. *Levo*-corydalmine (*l*-CDL), which exhibited micromolar affinity for D2DR in D2/CHO-K1 cell lines in this report and effectively alleviated bone cancer pain in our previous study, attenuated morphine tolerance in rats with chronic bone cancer pain at nonanalgesic doses. Furthermore, the intrathecal administration of *l*-CDL obviously attenuated morphine tolerance, and the effect was reversed by a D2DR agonist in mice. Spinal D2DR inhibition and *l*-CDL also inhibited tolerance induced by the MOR agonist DAMGO. *l*-CDL and a D2DR small interfering RNA (siRNA) decreased the increase in levels of phosphorylated Akt and MAPK in the spinal cord; these changes were abolished by a PI3K inhibitor. In addition, the activated Akt and MAPK proteins in mice exhibiting morphine tolerance were inhibited by a MOR antagonist. Intrathecal administration of a PI3K inhibitor also attenuated DAMGO-induced tolerance. Based on these results, *l*-CDL antagonized spinal D2DR to attenuate morphine tolerance by inhibiting PI3K/Akt-dependent MAPK phosphorylation through MOR. These findings provide insights into a more versatile treatment for morphine tolerance.

## Pain medicine: why blocking dopamine receptors alleviates morphine tolerance

By blocking dopamine receptors located in the spinal cord, a compound found in a traditional Chinese herbal medicine may help mitigate tolerance to morphine, a common problem among cancer patients who regularly take the opioid painkiller. A team led by Ji-Hua Liu and Bo-Yang Yu from China Pharmaceutical University in Nanjing had previously showed that inhibiting dopamine D2 receptors in spinal neurons prevented mice from developing morphine tolerance, but it wasn’t clear why. They have now demonstrated that blocking D2 receptors prevents the relay of cellular signals from morphine-binding “μ-opioid” receptors to mediators of drug tolerance. *Levo*-corydalmine, a compound isolated from the Asian Corydalis plant, binds and inhibits D2 receptors. When administered directly into the spinal cords of mice and rats, it blocked downstream signaling, reducing morphine tolerance.

## Introduction

Opioids are still extremely potent analgesics in the clinic, particularly for chronic severe pain, such as chronic bone cancer pain and other types of pain. However, repeated morphine treatments induce tolerance that contributes to the risk of developing drug dependence, addiction and tolerance, as dose escalation is required to maintain adequate analgesia.^1,2^ Despite the extensive research into the mechanism of morphine tolerance in the past few decades,^3,4^ morphine tolerance is still a substantial clinical challenge.

Considerable progress has been made in determining the mechanisms underlying opioid tolerance. The desensitization, internalization and downregulation of MOR,^4^ or heterodimerization with other receptors^5^ causes opioid receptor-mediated adaptive changes in the spinal cord to participate in the development of morphine tolerance.^4,6^ Moreover, chronic morphine exposure also enhances excitatory synaptic transmission by releasing chemokines, substance P (SP), glutamate (Glu), and other substances^6^ or directly activating glial cells in the spinal cord,^7–9^ leading to the activation of Ca^2+^-sensitive proteins, such as PKC γ, and MAPK to further enhance the excitability of spinal neurons.^10^ All these changes potentially contribute to the development of morphine tolerance. However, the intracellular processes that initiate the activation of multiple proteins after chronic morphine treatment remain unclear.

Based on accumulating evidence, D2DR, a G protein-coupled dopamine receptor, was reported to be involved in morphine-induced modulation of nociception. Activation^11–13^ and knockout of D2DR^14^ were both reported to potentiate the effect of opioid analgesia. Intraperitoneal injections of a D2DR antagonist^15,16^ and D2DR agonist^15,17^ were also both reported to attenuate morphine tolerance. As shown in our previous studies, blockade of D2DR in the spinal cord alleviates morphine tolerance.^18^ However, the exact mechanism by which D2DR blockade attenuates morphine tolerance remains to be elucidated. Therefore, in the present study, the mechanism underlying the effect of the blockade of spinal D2DR on regulating chronic morphine tolerance was further explored.

Surprisingly, *l-*CDL, which exerted a strong analgesic effect on chronic bone cancer pain in our previous study,^19^ attenuated morphine tolerance in rats with bone cancer pain at nonanalgesic doses. *l*-CDL is a natural product that was originally isolated from the traditional Chinese analgesic herb *Corydalis yanhusuo* W.T. Wang and is also a major metabolite of *l-*THP, which has been used in the clinic in China for over 50 years to alleviate pain.^20^ *l-*CDL is a tetrahydroprotoberberine that shows high affinity for dopamine receptors and has a variety of pharmacological activities without notable side effects.^21,22^ This study provides the first evidence that *l-*CDL effectively attenuated chronic morphine tolerance via D2DR antagonism in the spinal cord and has been considered a potential candidate for attenuating morphine tolerance.

## Materials and methods

### Materials

Morphine was obtained from Shenyang First Pharmaceutical Factory (Shengyang, China). Quinpirole, sulpiride, SCH-23390, DAMGO, CTOP, naltrindole and η-BNI were purchased from Tocris Bioscience (Ellisville, MO, USA). LY 294002 was purchased from Beyotime Biotechnology (Shanghai, China). The anti-glyceraldehyde 3-phosphate dehydrogenase (GAPDH) antibody was purchased from Sigma-Aldrich (St. Louis, MO, USA). The anti-phospho-p38 MAPK, anti-p38 MAPK, anti-phospho-p44/42 MAPK (ERK1/2), anti-p44/42 MAPK (ERK1/2), anti-phospho-SAPK/JNK, anti-SAPK/JNK, anti-phospho-Akt, and anti-Akt antibodies were purchased from Cell Signaling Technology (Beverly, MA, USA). Secondary antibodies used for western blotting were obtained from Cell Signaling Technology (Beverly, MA, USA). Secondary antibodies used for immunofluorescence were purchased from Jackson ImmunoResearch Laboratories Inc. (West Grove, PA, USA). The fluorescent dye fluo-8 was obtained from Invitrogen (Carlsbad, CA, USA). Ham’s F12 medium, zeocin, hygromycin B and HBSS and heat-inactivated fetal bovine serum were obtained from Gibco (Gaithersburg, MD, USA).

The siRNA targeting D2DR and its nonspecific oligonucleotide were synthesized by GenePharma Co. (Shanghai, China). Their sequences were: sense: A 5′-CUGUCAUGAUCGCCAUUGUtt-3′, B 5′-GAAGUC-UAAUGGGAGUUUCtt-3′, C 5′-CGCACAUCCUGAAUAUACAtt-3′, antisense: A 5′-ACAAUGGCGAUCAUGACAGtt-3′, B 5′-GAAACUC-CCAUUAGACUUCtt-3′, C 5′-UGUAUAUUCAGGAUGUGCGtt-3′; nonspecific oligonucleotide control, sense: 5′-UUCUCCGAACGUGUC-ACGUtt-3′, antisense: 5′-ACGUGACACGUUCGGAGAAtt-3′. Thirty-three micrograms of siRNA were diluted with 82.5 μl of RNA-free water, and this solution was further diluted with 82.5 μl of a 10% glucose solution. The solution was mixed by gentle vortexing or by pipetting up and down. Then, 49.5 μg of polyethyleneimine (PEI) were diluted with 82.5 μl of RNA-free water, and subsequently diluted with 82.5 μl of a 10% glucose solution. The siRNA solution and PEI solution were mixed and incubated 15 min at RT before use.^23,24^

### Animals

Adult male Institute of Cancer Research mice weighing 18–22 g at 8–10 weeks of age, Sprague–Dawley female rats weighing 180–220 g and 60–80 g were purchased from the Experimental Animal Center of Yangzhou University (Jiangsu province, China). Mice and rats were allowed free access to food and water and housed in room with a 12 h light/dark cycle at 22 °C. The mice and rats were randomly allocated to different groups by age and body weight. The behavioral tests were performed in a blinded manner, and the mice and rats were humanly euthanized with an injection of 100 mg/kg pentobarbital. The study was approved by the Animal Experimentation Ethics Committee of China Pharmaceutical University and performed in accordance with the guidelines of the International Association for the Study of Pain.

### Model of bone cancer pain induced by an intratibial inoculation of walker 256 mammary gland carcinoma cells

Walker 256 mammary gland carcinoma cells (5 × 10^6^ cells/ml, 0.5 ml) were implanted into rats weighing 60–80 g via an intraperitoneal injection. Five to seven days later, the ascites were extracted and centrifuged at 2000 rpm for 6 min; then, the sediment was washed with 0.01 M PBS two times and diluted to a density of 1 × 10^5^ cells/μl with sterile 0.01 M PBS. The cell suspension was incubated on ice until it was injected into tibia of rats weighing 180–220 g. The model of Walker 256 ascites tumor cell-induced bone cancer pain was generated as described in a previous study.^19^ Briefly, rats were anesthetized with sodium pentobarbital (50 mg/kg, i.p.) and the tibia head in the left leg was exposed with minimal damage. Tumor cells (5 μl) were slowly injected into the medullary cavity of the tibia, and 5 μl of PBS were injected into the medullary cavity of the controls. Bone wax was applied for 3 min to prevent the escape of tumor cells from the bone when the syringe was removed. The injection site was closed using dental cement.

### Behavioral assessment of antinociceptive effects and chronic morphine tolerance tests

The antinociceptive effects on mice were tested by performing the tail-flick test using previously described methods (52 °C water bath).^25^ A cut off time of 10 s was established to avoid tissue damage. The responses were calculated as a percentage of the maximal possible effect (% MPE) using the following formula: 100% × [(drug response time**—**basal response time)/(10 s**—**basal response time)] = %MPE.^18^ Morphine (10 μg/10 μl, i.t.) was administered to mice daily for 7 days to induce morphine tolerance and the analgesic effect was measured 30 min after each injection.

### Behavioral assays for bone cancer-related pain

Rats were placed in a large transparent plastic mesh cage for 15–30 min and allowed to acclimate to the environment before the testing. Mechanical allodynia was measured using Von Frey filaments (Woodland Hills, Los Angeles).^26^ An ascending series of Von Frey filaments (1.4, 2.0, 4.0, 6.0, 8.0, 10.0, 15.0, and 26.0 g) were used to stimulate the hind paw of rats with logarithmically incremental increases in stiffness, and each Von Frey filament was held on the animal’s paw for approximately 5 s. A positive response was defined as a withdrawal of the hind paw upon stimulation. Whenever a positive response to a stimulus occurred, the next lower von Frey filament was applied, and whenever a negative response occurred, the next higher filament was applied. Each rat was tested at least three times and the average threshold was measured. The experimenters were blinded to the treatment. Tests were conducted 0.5 h after the morphine treatment to examine the immediate effect of *l*-CDL on morphine tolerance in TCI rats.

### Intrathecal injection procedure

The mouse was placed in a prone position and the midpoint between the tips of the iliac crest was located. Intrathecal injections were performed by introducing a lumbar puncture at the intervertebral space of L4–5 or L5–6 using a stainless steel needle (30 G). The proper injection would be accompanied by a tail-flick. The injection did not affect the baseline threshold of the rats.^27^

### Western blot

The levels of the phosphorylated MAPK, GFAP, and IBA1 proteins in the spinal cord tissues (spinal cord segments at L4–L6) were analyzed using previously described methods.^18^ Tissues were collected and lysed in RIPA buffer. Then, whole lysates were prepared, and lysates containing 50 μg of proteins were separated on sodium dodecyl sulfate–polyacrylamide gels and transferred onto polyvinylidene difluoride membranes. Membranes were blocked with 5% BSA for 2 h at room temperature and incubated overnight with primary antibodies at 4 °C. Then, membranes were washed with 0.1% TBST three times (10 min each) and incubated with secondary antibodies for 2 h at room temperature. Signals were ultimately detected using ECL reagents (PerkinElmer, Waltham, MA, USA). Data were analyzed using a Molecular Imager (Gel Doc^TM^ XR, 170–8170) and the associated Quantity One-4.6.5 software (Bio-Rad Laboratories, USA).

### Cell culture

The D2/CHO-K1 cell line was cultured in Ham’s F12 medium supplemented with 10% FBS, 200 μg/ml zeocin, and 100 μg/ml hygromycin B. D2/CHO-K1 cells were plated in a 384-well microplate at a density of 1.5 × 10^4^ cells/well (20 μl/well). Cells were incubated at 37 °C with a 5% CO_2_ atmosphere and 95% humidity for 18 h.

### Monitoring the Ca^2+^ response using image plate reader (FLIPR)

The conditioned medium of the cells cultured in the 384-well microplate was removed and replaced with loading buffer containing 4 μM fluo-8 dye. For agonist treatments, the cells were incubated with 20 μl of the dye for 1 h. Then, the plate was transferred to the chamber of a fluorescence laser plate reader. Fluorescence was recorded for 20 s to establish the baseline, and then 10 μl of the 5× agonist or compound were added to the corresponding wells. For antagonist tests, the cells were incubated with 20 μl of the dye and 10 μl of solutions containing the compound for 1 h. Fluorescence was recorded for 20 s to establish the baseline, and then 12.5 μl of the (5× EC_80_) agonist solutions were added to the corresponding wells. The cells were tested for a total of 120 s.

### Statistical analysis

SPSS version 15 (SPSS Inc., Chicago, IL, USA) and GraphPad Prism software (Version 6.0; GraphPad Software Inc., San Diego, CA) were used to conduct the statistical analyses. Data were statistically evaluated using one-way analysis of variance (ANOVA) and the two-way ANOVA followed by Bonferroni’s post hoc tests. The results are presented as the means ± standard errors of three independent experiments performed in triplicate. The results described as significant are based on a criterion of *P* < 0.05.

## Results

### Intrathecal administration of *l-*CDL attenuates morphine tolerance

**Fig. 1:**
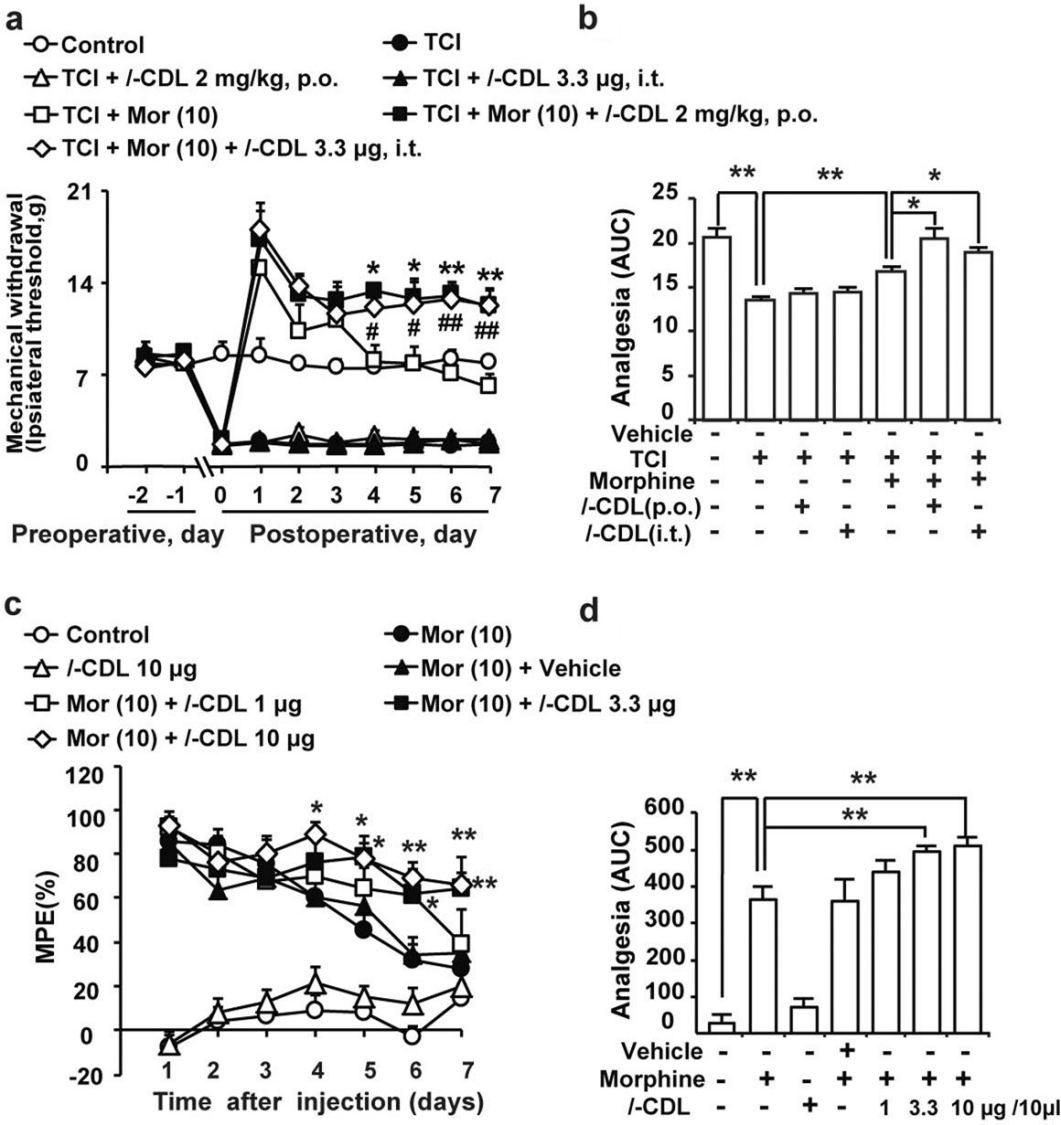
Intrathecal administration of *l-*CDL attenuates morphine tolerance and antinociceptive effects. Rats and mice were examined daily with Von Frey filaments and the tail-flick assay, respectively. Data are presented as percentages of the maximal possible effect (% MPE). The analgesic effect of morphine was decreased after the chronic morphine treatment, indicating the development of morphine tolerance. **a** *l-*CDL attenuated morphine tolerance in TCI rats at nonnarcotic analgesic doses. **b** The analgesia was further reported as area under the curve (AUC) units. Data are presented as means ± SE. *n* = 6, ^*^P < 0.05, ^**^P < 0.01, ^#^*P* < 0.05, ^##^*P* < 0.01 compared with the morphine group. **c** *l*-CDL attenuated the development of morphine tolerance and did not affect the pain threshold in naïve mice. **d** The analgesia was further reported in AUC units. Data are presented as means ± SE. *n* = 12, ^*^P < 0.05, ^**^P < 0.01, compared with the morphine group

*l-*CDL attenuated the development of morphine tolerance in rats with chronic bone cancer pain induced by tibia bone cavity tumor cell implantation (TCI). The Von Frey filament test showed that TCI rats developed tolerance to the analgesic effect of morphine after chronic morphine treatment (10 μg/20 μl) for 7 days. Both intragastric administration of *l*-CDL (2 mg/kg) and intrathecal administration of *l*-CDL (3.3 μg/20 μl) (15 min before the morphine treatment) markedly attenuated the development of morphine tolerance, but did not exert effects on the pain threshold in TCI rats (Fig. 1a, b). Furthermore, normal mice also developed tolerance to the analgesic effect of morphine after chronic treatment. Co-administration of *l-*CDL (1, 3.3, 10 μg/10 μl, i.t., 15 min before the morphine treatment) significantly attenuated the development of chronic morphine tolerance without affecting basal pain perception, and even 1% DMSO did not affect the development of morphine tolerance. The MPE in the morphine treatment group on day 7 was 27.96 ± 14.66%, while mice that were co-administered *l*-CDL (1, 3.3, 10 μg/10 μl, i.t.) exhibited MPE values of 39.28 ± 15.46%, 64.22 ± 14.42% and 65.80 ± 5.79%, respectively. The analgesia was further presented in area under the curve (AUC) units (Fig. 1c, d).

### An antagonist of spinal D2DR, but not D1DR, attenuates morphine tolerance and a D2DR agonist reverses the inhibitory effect of *l-*CDL on morphine tolerance in mice

**Fig. 2:**
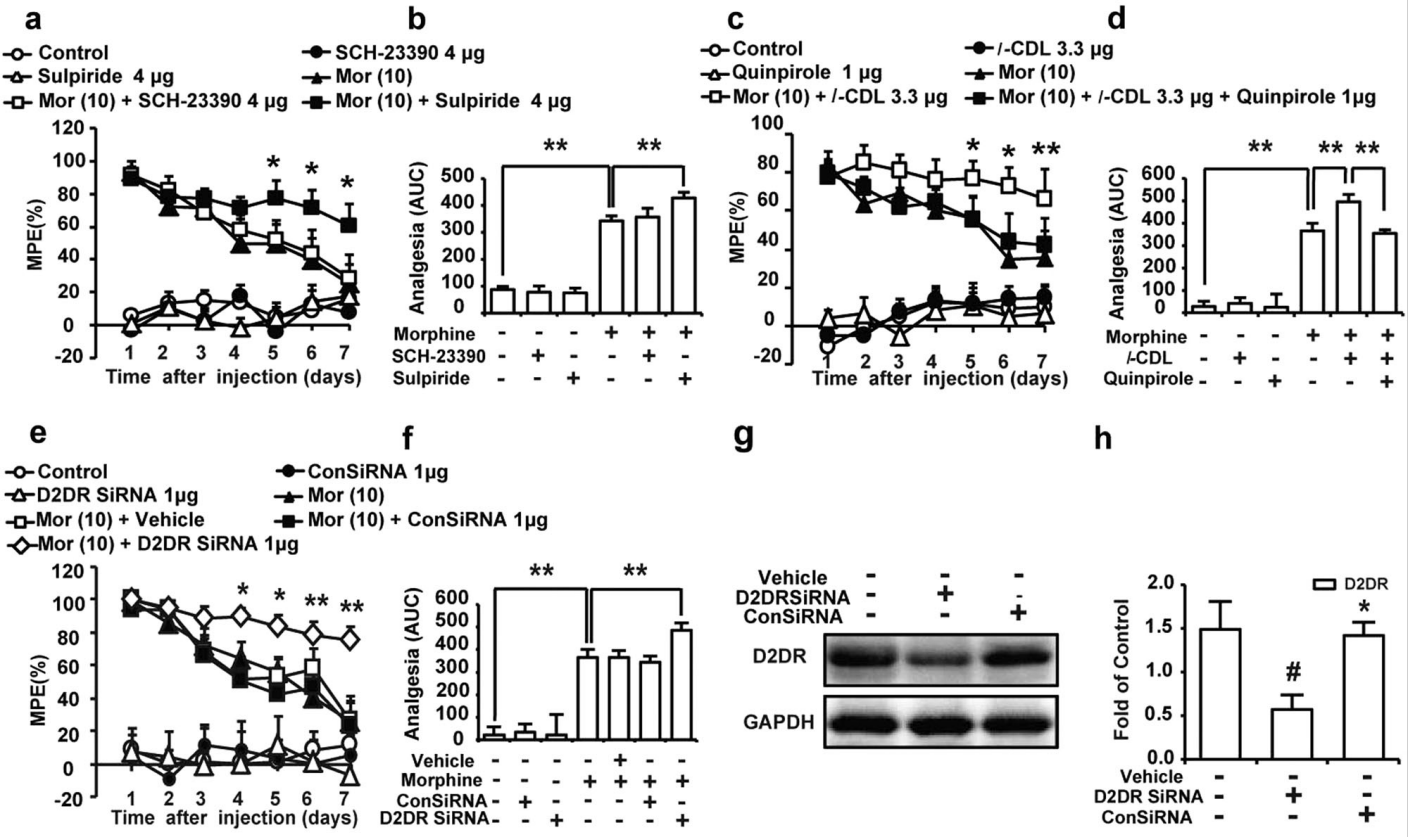
Antagonism of spinal D2DR, but not D1DR, attenuates morphine tolerance and a D2DR agonist reverses the inhibitory effects of *l-*CDL on morphine tolerance in mice. **a**, **b** Intrathecal administration of the D2DR antagonist sulpiride (4 μg/10 μl) attenuated morphine tolerance, while the D1DR antagonist SCH-23390 (4 μg/10 μl) did not attenuate morphine tolerance. Neither sulpiride nor SCH-23390 affected the pain threshold of naïve mice. The analgesia was further reported in area under the curve (AUC) units. Data are presented as means ± SE. *n* = 12, ^*^P < 0.05, ^**^P < 0.01, compared with the morphine group. **c**, **d** Intrathecal administration of the D2DR agonist quinpirole (1 μg/10 μl) abolished *l*-CDL (3.3 μg/10 μl)-induced inhibition of morphine tolerance in mice. The analgesia was further reported as area under the curve (AUC) units. Data are presented as means ± SE. *n* = 12, ^*^P < 0.05, **P <0.01, compared with the group treated with the combination of morphine, *l*-CDL and quinpirole. **e**, **f** Multiple daily intrathecal injections of the D2DR siRNA for 7 days effectively attenuated the development of morphine tolerance, while the nonspecific oligonucleotide had no effect. Neither the D2DR siRNA nor the nonspecific oligonucleotide altered the pain threshold of naïve mice. Data are presented as means ± SE. *n* = 12, ^*^P < 0.05, ^**^P < 0.01, compared with the morphine group. **g**, **h** Intrathecal administration of the D2DR siRNA for 7 days obviously reduced the expression of D2DR in the spinal cord. Representative western blot bands and a summary of the data are shown. Data are presented as means ± SE. ^n^ = 4, ^#^*P* < 0.05, ^##^*P* < 0.01, compared with the vehicle; ^*^P < 0.05, ^**^P < 0.01, compared with the siRNA group

The D2DR antagonist sulpiride (4 μg/10 μl, i.t., 15 min before the morphine treatment) markedly attenuated morphine tolerance, similar to *l*-CDL, while the dopamine D1 receptor (D1DR) antagonist SCH-23390 (4 μg/10 μl, i.t.) did not attenuate morphine tolerance. As expected, neither antagonist alone altered the pain threshold of mice (Fig. 2a, b). Moreover, the intrathecal administration of the D2DR agonist quinpirole (1 μg/10 μl, 15 min before the *l-*CDL treatment) abolished the inhibitory effects of *l-*CDL (3.3 μg/10 μl, i.t., 15 min before the morphine treatment) on morphine tolerance (Fig. 2c, d). A D2DR siRNA was used to further confirm the effect of spinal D2DR inhibition on morphine tolerance. One microgram of the RNA was dissolved in 1.5 μg of PEI. Each mouse in the D2DR siRNA group received multiple daily intrathecal injections of the D2DR siRNA solution (1 μg/10 μl) for 7 days, and mice in the control RNA (conRNA) group received multiple daily intrathecal injections of a nonspecific oligonucleotide (1 μg/10 μl). On the seventh day, the spinal cord was collected 30 min after the siRNA treatment, and proteins were extracted from spinal lumbar segments and subjected to western blot analyses. The intrathecal administration of D2DR siRNA significantly reduced the levels of the D2DR protein by 61.6% and 59.9%, respectively, compared to the vehicle and nonspecific oligonucleotide groups (Fig. 2g, h). After treatment with the siRNA for 7 days, mice received a continuous intrathecal infusion of morphine (10 μg/10 μl) beginning on day 8 for 7 days, and the analgesic effect was measured 30 min after each injection. The intrathecal administration of the D2DR siRNA also effectively attenuated the development of morphine tolerance, while the nonspecific oligonucleotide did not exert this effect. The MPE in the morphine treatment group on day 7 was 27.96 ± 14.66%, mice preadministered the siRNA and conRNA (1 μg/10 μl, i.t.) exhibited MPE values of 75.56 ± 7.94% and 23.17 ± 13.97%, respectively, the analgesia was further described in AUC units (Fig. 2e, f).

### *l-*CDL shows micromolar affinity for D2DR

**Fig. 3:**
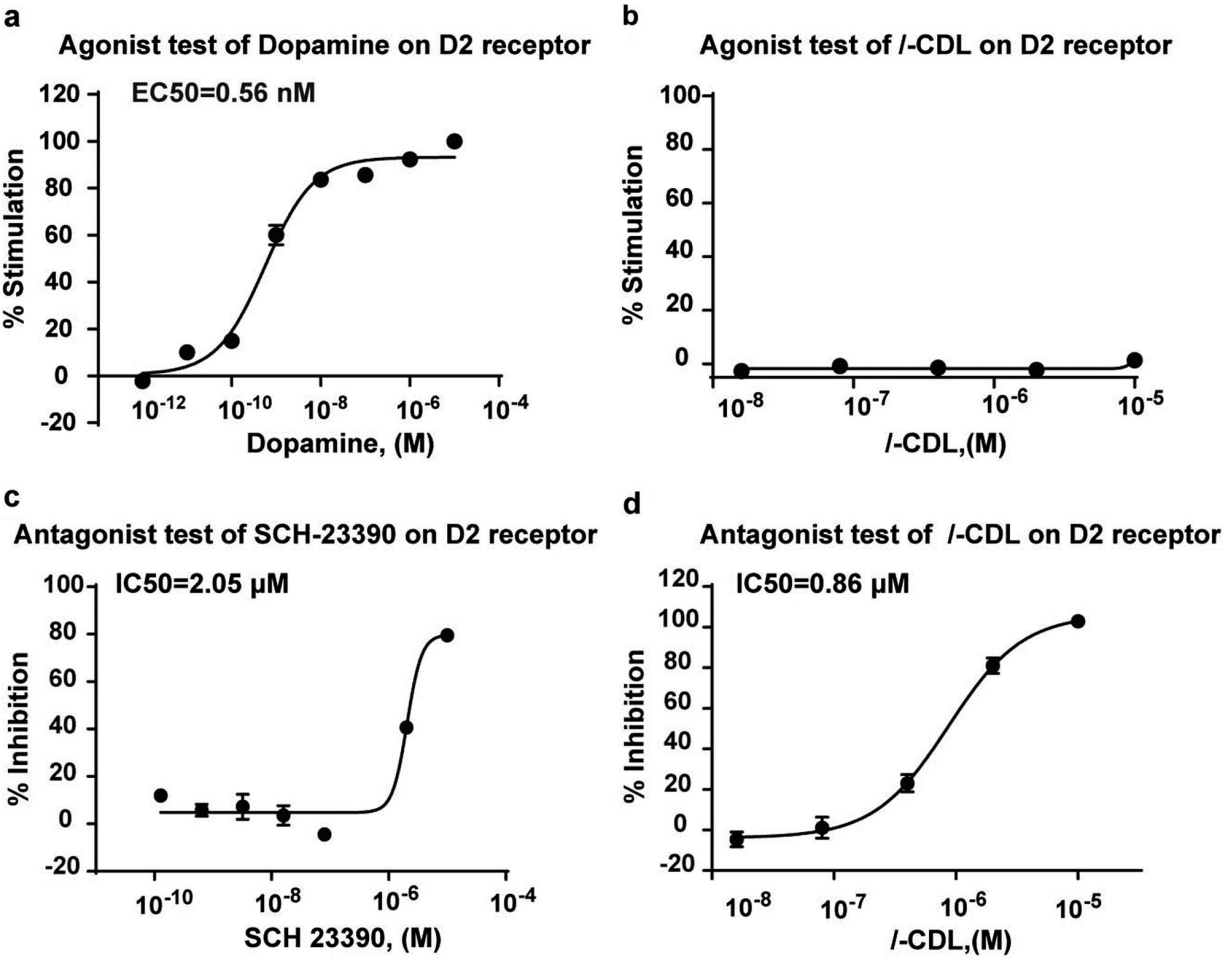
*l-*CDL shows micromolar affinity for D2DR. **a** The EC50 of dopamine-induced Ca^2+^ mobilization in D2/CHO-K1 cells was 0.56 nM. **b** *l-*CDL did not activate D2DR to induce Ca^2+^ mobilization in D2/CHO-K1 cells. **c** The D2DR antagonist SCH-23390 inhibited dopamine-induced Ca^2+^ mobilization in D2/CHO-K1 cells with an IC50 of 2.05 μM. **d** *l-*CDL antagonized dopamine-induced Ca^2+^ mobilization in D2/CHO-K1 cells with an IC50 of 0.86 μM (*n* = 4)

The activity of *l-*CDL toward D2DR was further explored in this article. *l*-CDL inhibited dopamine-induced Ca^2+^ mobilization in D2/CHO-K1 cells, with a half maximal inhibitory concentration (IC50) of 0.86 μM (Fig. 3d), while it did not activate D2DR to induced Ca^2+^ mobilization (Fig. 3b), indicating that *l*-CDL exhibited micromolar affinities for D2DR, similar to other D2DR antagonists, *l-*THP and DHCB.^28^ The dopamine receptor agonist dopamine and D2DR antagonist SCH-23390 were used as positive controls. Dopamine induced Ca^2+^ mobilization in D2/CHO-K1 cells with a half maximal effective concentration (EC50) of 0.56 nM (Fig. 3a), and the D2DR receptor antagonist SCH-23390 inhibited dopamine-induced Ca^2+^ mobilization in D2/CHO-K1 cells with an IC50 of 2.05 μM (Fig. 3c).

### *l-*CDL antagonizes spinal D2DR to attenuate morphine tolerance in mice in a MOR-dependent manner

**Fig. 4:**
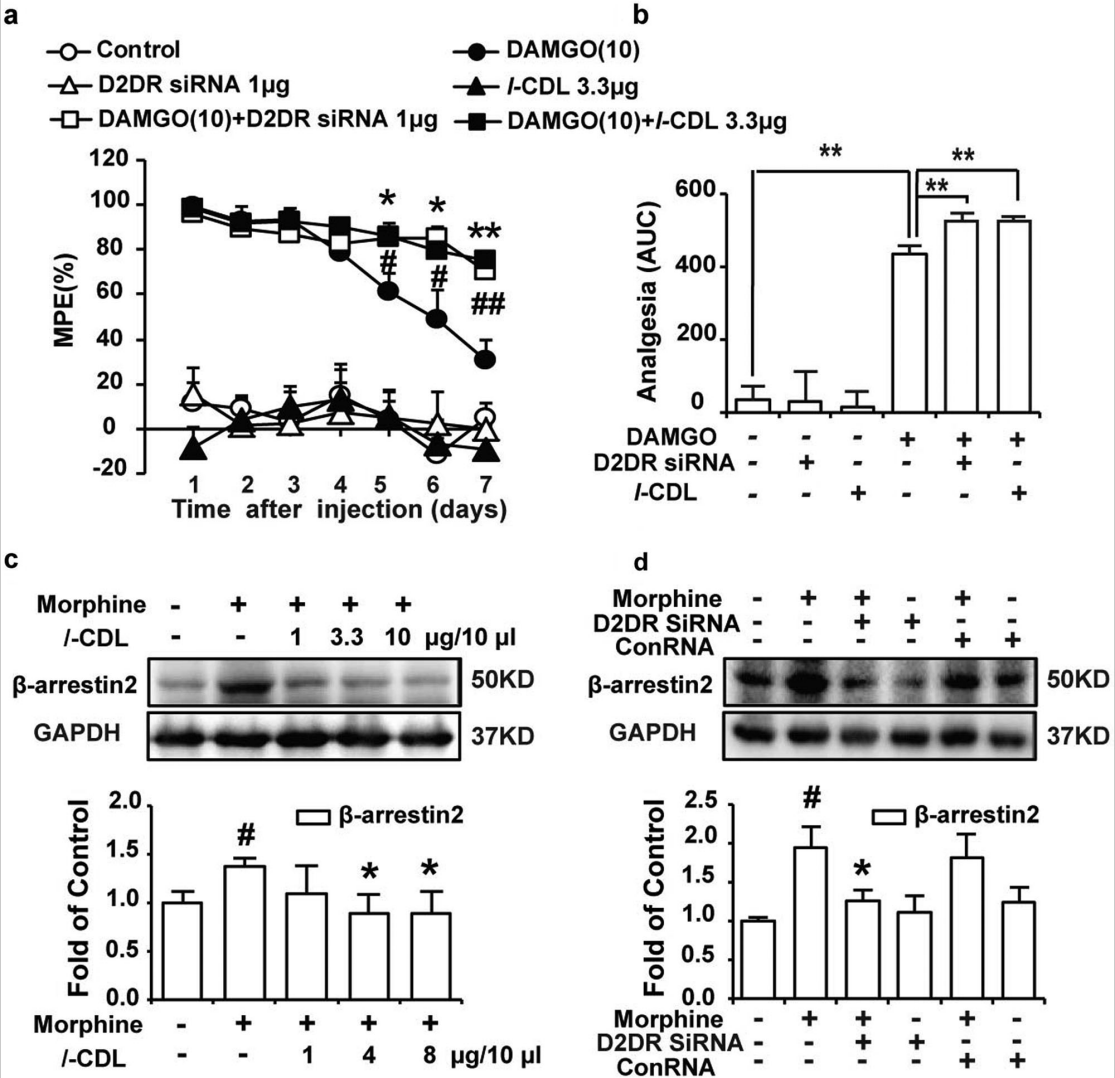
l-CDL antagonizes spinal D2DR to attenuate morphine tolerance in mice in a MOR-dependent manner. **a**, **b** Intrathecal administration of *l-*CDL and the D2DR siRNA attenuated tolerance induced by the chronic DAMGO treatment. Data are presented as means ± SE. *n* = 12, ^#^*P* < 0.05, ^##^*P* < 0.01, comparison between groups treated with the combination of *l*-CDL and DAMGO and DAMGO alone; ^*^P < 0.05, ^**^P < 0.01, comparison of groups treated with the combination of the D2DR siRNA and DAMGO and DAMGO alone. Mice were examined daily using the tail-flick assay. Data are presented as percentages of the maximal possible effect (% MPE). **c**, **d** The expression of β-arrestin2 was upregulated in the spinal cord, and the intrathecal administration of both *l*-CDL and the D2DR siRNA decreased the upregulation of β-arrestin2 expression in the spinal cord. Data are presented as means ± SE. *n* = 4, ^#^*P* < 0.05, ^##^*P* < 0.01, compared with the vehicle; ^*^P < 0.05, ^**^P < 0.01, compared with the morphine group

Morphine is known to exert its analgesic effect mainly by activating the MOR protein encoded by the MOR-1 gene. MOR-1 knockout mice do not display morphine analgesia and tolerance.^29,30^ The mechanism by which spinal D2DR blockade with its antagonist effectively attenuates chronic morphine tolerance is probably related to the regulation of MOR activity.^18^ DAMGO, a selective MOR agonist was administered to the mice and the antinociceptive effects were measured using the tail-flick test to further determine the involvement of MOR in D2DR-mediated inhibition of morphine tolerance. As shown in Fig. 4a, b, the antinociceptive effects of DAMGO were dramatically decreased after the chronic administration of DAMGO (10 μg/10 μl, i.t.) for 7 days, indicating the development of tolerance. Intrathecal administration of *l-*CDL (3.3 μg) and the D2DR siRNA (1 μg) (15 min before the DAMGO treatment) obviously attenuated DAMGO-induced tolerance (Fig. 4a, b). β-Arrestin2 is a multifunctional protein that participates in GPCR signaling and is involved in the desensitization of MOR.^31^ Mice lacking β-arrestin2 display reduced tolerance to these antinociceptive effects,^32,33^ and in β-arrestin2 knockout mice, do not develop tolerance to a chronic morphine treatment,^33^ suggesting that β-arrestin2 is required for the development of morphine tolerance. D2DR was reported to recruit β-arrestin2 and increase its expression.^34,35^ We wondered whether D2DR increased the expression of β-arrestin2 to promote the development of morphine tolerance. The expression of β-arrestin2 was upregulated in the spinal cord after chronic morphine treatment, consistent with other reports.^36^ Both intrathecal administration of the D2DR siRNA and *l-*CDL decreased the increased expression of β-arrestin2 (Fig. 4c, d).

### *l-*CDL antagonizes spinal D2DR to attenuate morphine tolerance in mice by inhibiting the PI3K/Akt signaling pathway in a MOR-dependent manner

**Fig. 5:**
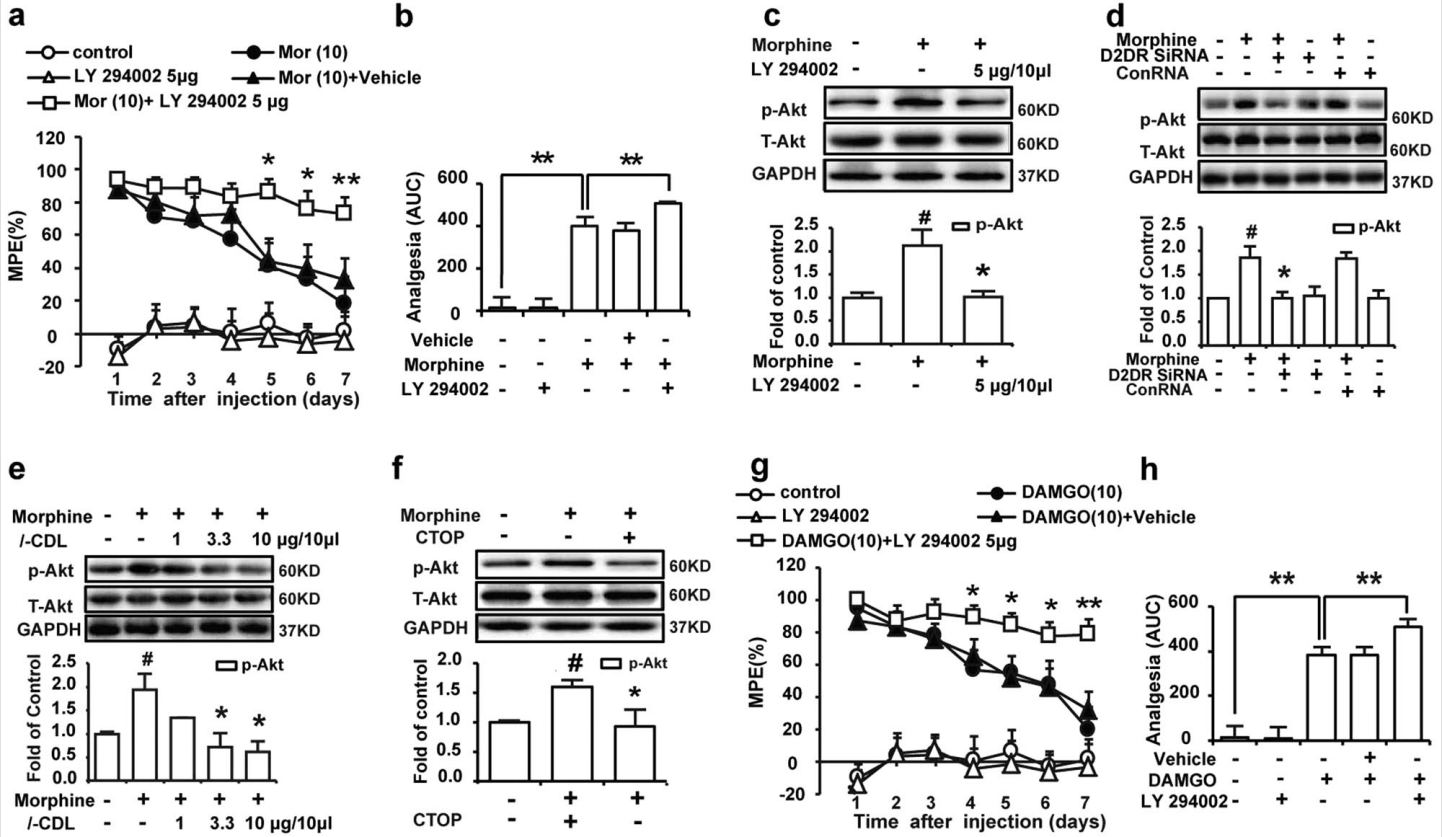
*l-*CDL antagonizes spinal D2DR to attenuate morphine tolerance in mice by inhibiting PI3K/Akt signaling in a MOR-dependent manner. **a**, **b** Intrathecal administration of the PI3K inhibitor LY 294002 (5 μg/10 μl) attenuated morphine tolerance, while LY 294002 (5 μg/10 μl) alone did not affect the pain threshold of naïve mice. The analgesia was further reported as area under the curve (AUC) units. Data are presented as means ± SE. *n* = 12, ^*^P < 0.05, ^**^P < 0.01, compared with the morphine group. **c** Intrathecal administration of LY 294002 (5 μg/10 μl) reduced the increase in p-Akt levels in the spinal cord. **d**, **e** Intrathecal administration of the D2DR siRNA and *l*-CDL reduce p-Akt levels in the spinal cord, while intrathecal administration of the D2DR conRNA had no effect. **f** Intrathecal administration of the MOR antagonist CTOP (1 ng/10 μl) reversed the increased p-Akt levels in the spinal cord. Data are presented as means ± SE. *n* = 4, ^#^*P* < 0.05, ^##^*P* < 0.01, compared with the control group; ^*^P < 0.05, ^**^P < 0.01, compared with the morphine group. **g**, **h** Intrathecal administration of the PI3K inhibitor LY 294002 attenuated DAMGO-induced tolerance. All spinal cords were collected on day 7, 30 min after chronic morphine exposure. Data are presented as means ± SE. *n* = 12, ^*^P < 0.05, ^**^P < 0.01, compared with the DAMGO group

Inhibition of spinal PI3K/Akt signaling significantly attenuates morphine tolerance,^37^ and activation of spinal D2DR activates PI3K/Akt signaling through both G protein-dependent and -independent mechanisms.^38,39^ We, therefore, challenged the mice with morphine and the specific PI3K inhibitor LY 294002; LY 294002 (5 μg/10 μl, i.t., 15 min before the morphine treatment) significantly attenuated morphine tolerance, and the inhibitor alone did not change the pain threshold of mice (Fig. 5a, b). LY 294002 (5 μg/10 μl, i.t.) also reduced the increased levels of p-Akt in the spinal cord (Fig. 5c). Antagonism of spinal D2DR with its siRNA (1 μg/10 μl, i.t.) and intrathecal administration of *l-*CDL (3.3, 10 μg/10 μl, i.t.) effectively reduced the morphine tolerance-induced increase in p-Akt levels (Fig. 5d, e). Thus, *l-*CDL antagonized spinal D2DR and inhibited its downstream PI3K/Akt signaling to attenuate morphine tolerance. We further explored whether antagonism of spinal D2DR by *l*-CDL attenuated morphine tolerance through PI3K/Akt signaling in a MOR-dependent manner. The intrathecal administration of the PI3K inhibitor LY294002 (5 μg/10 μl, i.t.) also attenuated DAMGO-induced tolerance. The increased level of p-Akt induced by morphine tolerance was attenuated by co-administration of the selective MOR antagonist CTOP (1 ng/10 μl, i.t., 15 min before the morphine treatment), but not the selective κ opioid receptor antagonist η-BNI (1 ng/10 μl, i.t.) or the selective δ opioid receptor antagonist naltrindole (1 ng/10 μl, i.t.) (Fig. 5f–h and Supplementary Figs. 1a, e). Chronic DAMGO treatment also increased the levels of p-Akt, an effect that was inhibited by LY 294002 (5 μg/10 μl, i.t.) (Supplementary Fig. 2a).

### *l-*CDL antagonizes spinal D2DR to attenuate morphine tolerance in mice through the PI3K/Akt-MAPK signaling pathway in a MOR-dependent manner

**Fig. 6:**
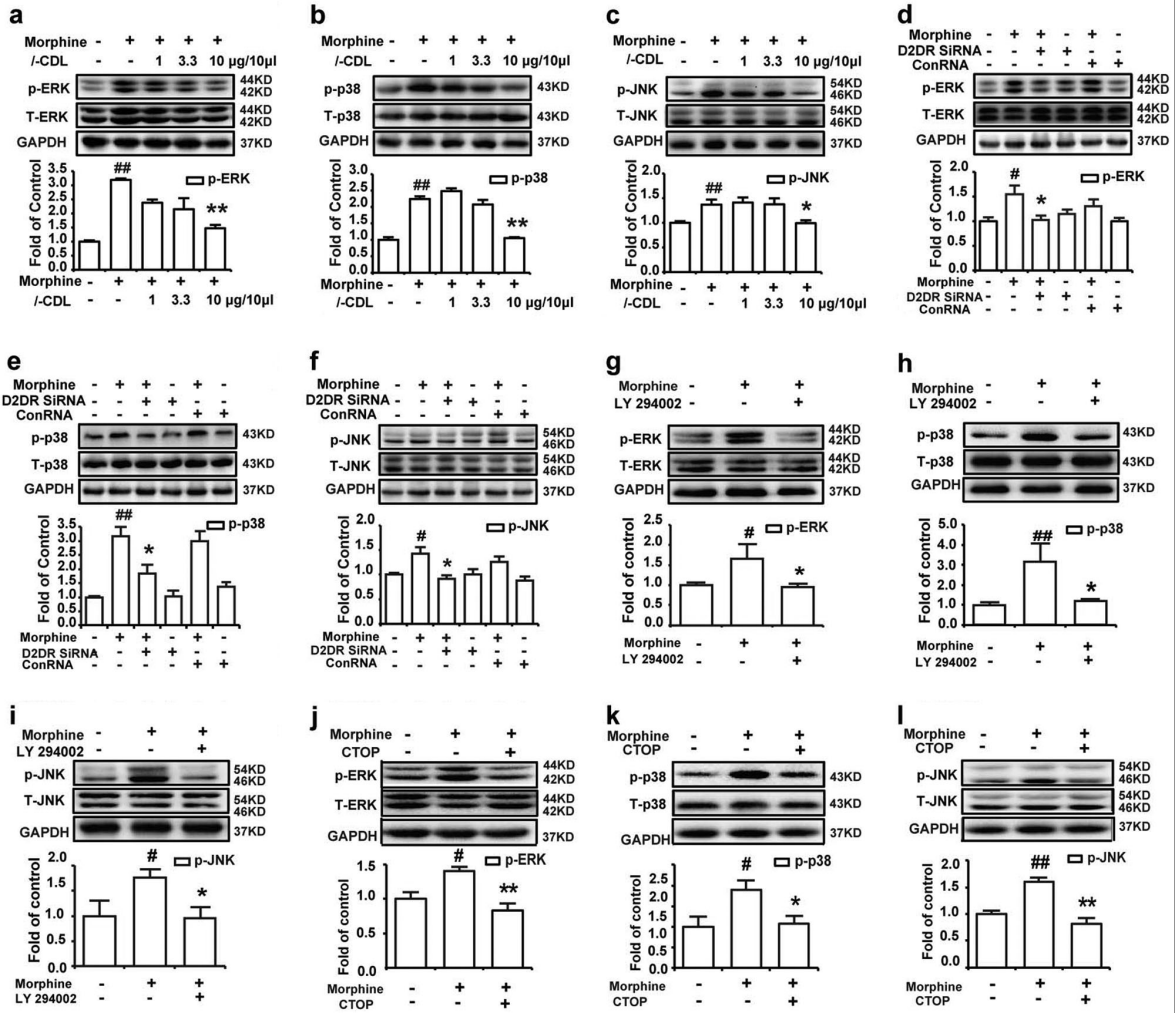
*l-*CDL antagonizes spinal D2DR to attenuate morphine tolerance in mice through PI3K/Akt-MAPK signaling in a MOR-dependent manner. **a**–**c** Western blots showing increased levels of p-ERK1/2, p-p38, and p-JNK in the spinal cord after chronic morphine treatment, changes that were significantly decreased by the intrathecal administration of *l-*CDL. **d**–**f** Mice receiving a spinal D2DR siRNA injection with a morphine infusion showed a significant reduction in the levels of p-ERK1/2, p-p38, and p-JNK in the spinal cord. **g**–**i** Inhibition of spinal PI3K with its inhibitor LY 294002 also decreased the levels of p-ERK1/2, p-p38, and p-JNK in the spinal cord. **j**–**l** Intrathecal administration of the MOR antagonist CTOP (1 ng/10 μl) reversed the increased levels of p-ERK1/2, p-p38, and p-JNK in the spinal cord. Representative western blot bands and a summary of the data are shown. All spinal cords were collected on day 7, 30 min after chronic morphine exposure. Data are presented as means ± SE. *n* = 4, ^#^*P* < 0.05, ^##^*P* < 0.01, compared with the control group; ^*^P < 0.05, ^**^P < 0.01, compared with the morphine group

Chronic morphine treatment increased the levels of phosphorylated extracellular signal-regulated kinase (ERK1/2), c-JUN N-terminal kinase (JNK) and p38 MAPK in the spinal cord, which were reported to promote the development of morphine tolerance.^40^ Here, the intrathecal administration of both *l*-CDL and the D2DR siRNA significantly reduced the increased levels of p-ERK1/2, p-JNK and p-p38 in the spinal cord (Fig. 6a–f). The MAPK and PI3K/Akt signaling pathways interact with each other to perform different functions.^41^ Similar to reports that D2DR activates p-ERK1/2 through PI3K in the opossum kidney,^42^ intrathecal administration of LY 294002 (5 μg/10 μl) also reduced the levels of p-ERK1/2, p-JNK and p-p38 in the spinal cord in the present study (Fig. 6g–i) indicating that *l-*CDL antagonized spinal D2DR to attenuate morphine tolerance by inhibiting PI3K/Akt-dependent MAPK phosphorylation. Furthermore, the increased levels of p-MAPK were attenuated by co-administration of the selective MOR antagonist CTOP (1 ng/10 μl, i.t., 15 min before the morphine treatment), but not the selective κ and δ opioid receptor antagonists η-BNI (1 ng/10 μl, i.t.) and naltrindole (1 ng/10 μl, i.t.), respectively (Fig. 6j–l and Supplementary Fig. 1b–d, f–h). A chronic DAMGO treatment also increased the p-MAPK levels, a change that was inhibited by LY 294002 (5 μg/10 μl, i.t.) (Supplementary Fig. 2b–d).

## Discussion

The present study is the first to show that specific blockade of spinal D2DR inhibited PI3K/Akt-mediated MAPK phosphorylation in a MOR-dependent manner to attenuate morphine tolerance in mice, which may represent a new mechanism to effectively attenuate morphine tolerance. A natural analgesic compound, *l*-CDL, significantly attenuated chronic morphine tolerance by acting as a D2DR antagonist in the spinal cord at nonanalgesic doses.

D2DR has been reported to be involved in morphine antinociception and tolerance. The D2DR agonist bromocriptine (i.p.)^17^ enhances the ability of the NMDA antagonist MK-801 to attenuate the development of morphine tolerance, while other researchers revealed that both the D2DR agonist quinpirole (i.p.)^15^ and D2DR antagonists sulpiride (i.p.)^15^ and eticloride (i.p.)^16^ decrease the development of morphine tolerance. The dorsal horn of the spinal cord is strongly implicated in the development of chronic morphine tolerance.^43^ As shown in our previous report, the expression of D2DR is upregulated in the spinal cord and spinal D2DR antagonism significantly attenuates morphine tolerance.^18^ Unfortunately, the exact mechanism by which spinal D2DR blockade modulates morphine tolerance remains unclear, and clinically approved drugs targeting D2DR have undesirable side effects.^44^ Targeting of a specific dopamine receptor using a gene silencing method such as siRNAs might be safe and useful,^45^ but it still has not been used in the clinic. In the current study, the natural compound *l*-CDL, which exerted a potent analgesic effect on alleviating bone cancer pain, significantly attenuated morphine tolerance by antagonizing spinal D2DR at nonnarcotic analgesic doses, and we further explored its underlying mechanism. Both intragastric and intrathecal administration of *l-*CDL were reported to attenuate chronic pain,^19,46,47^ and *l-*CDL (p.o.) did not affect the motor function of rats^19^ at analgesic doses in our previous study. In the present study, *l-*CDL (p.o.) did not affect the locomotor activities of mice at analgesic doses (Fig. 4). According to the in vitro results, *l-*CDL (50, 30, 10, 3, 1, 0.3, and 0.1 μM) did not affect the viability of primary cultures of spinal cord neurons (Fig. 3). As the analgesic doses are much higher than nonanalgesic doses, *l-*CDL did not exhibit spinal cord or nerve toxicity, even at analgesic doses, suggesting that *l*-CDL has great potential for attenuating morphine tolerance in the clinic.

Morphine predominantly binds to MOR and weakly binds to κ and δ opioid receptors,^48^ and morphine-induced antinociception and tolerance are absent in MOR knockout mice. As shown in our previous study, MOR forms complexes with D2DR during morphine tolerance, and spinal D2DR antagonism significantly decrease the amount of the complex,^18^ but we did not confirm whether MOR was involved in D2DR blockade-induced attenuation of tolerance. Here, antagonism of spinal D2DR and intrathecal administration of *l*-CDL attenuated tolerance through a mechanism mediated by MOR. β-Arrestin2 plays an important role in the desensitization of MOR, as β-arrestin2 deletion accelerates the rate of MOR resensitization and disruption of β-arrestin2-dependent receptor trafficking promotes MOR resensitization, thereby attenuating morphine tolerance.^31,49^ In this report, the expression of β-arrestin2 was upregulated in the spinal cord of rats with chronic morphine tolerance, and spinal administration of *l-*CDL and the D2DR siRNA significantly reduced the expression of β-arrestin2. Based on this result, blockade of spinal D2DR by *l-*CDL probably reduced the expression of β-arrestin2 to increase the resensitization of MOR.

One other important observation in this article is that antagonism of spinal D2DR attenuated morphine tolerance by inhibiting PI3K/Akt-dependent MAPK phosphorylation. Studies of the tyrosine kinase class of receptors (G protein-coupled receptors) showed that upon agonist binding, the GTP-bound Gα subunit dissociates from the Gβγ subunit. The Gβγ subunit then activates the PI3K/Akt pathway.^38^ D2DR was also reported to activate the PI3K/Akt signaling pathway in a G protein-independent manner.^50^ D2DR stimulates the formation of a protein complex composed of β-arrestin2, PP2A, and Akt to activate Akt-medicated signaling.^51^ The activation of PI3K/Akt signaling promotes the development of morphine tolerance,^37,52^ but researchers have not determined whether the PI3K/Akt signaling pathway is involved in morphine tolerance. The MAPK family, which includes extracellular signal-regulated protein kinase 1/2 (ERK1/2), p38 and c-Jun N-terminal kinase (JNK), is also involved in morphine tolerance.^7–9,53,54^ We recently reported increased levels of p-ERK1/2, p-JNK, and p-p38 in the spinal cord of animals displaying morphine tolerance that were reversed by an intrathecal administration of a D2DR antagonist. In this article, intrathecal administration of *l-*CDL and the D2DR siRNA also decreased the increased levels of phosphorylated Akt and MAPK in the spinal cord of animals displaying morphine tolerance.

The MAPK and PI3K/Akt signaling pathways were reported to interact with each other to perform different functions.^41^ In anti-CD3-treated T cells, the rat skeletal muscle cell line L6 and PDGF-treated cultured airway smooth muscle cells, a PI3K inhibitor was reported to block the activation of ERK.^55–57^ A PI3K inhibitor was also reported to inhibit platelet-activating factor-induced activation of MAPK in a murine macrophage cell line.^58^ Thus, we wondered whether PI3K/Akt signaling mediated MAPK activation in morphine tolerance. The intrathecal administration of the PI3K inhibitor LY 294002 abolished the increased levels of p-ERK1/2, p-JNK, and p-p38 in the spinal cord, indicating that antagonism of spinal D2DR attenuated morphine tolerance by altering PI3K/Akt-MAPK signaling.

Furthermore, MOR was involved in spinal D2DR blockade-induced inhibition of PI3K/Akt-MAPK signaling. MOR-triggered activation of PI3K/Akt is involved in the development of morphine tolerance,^37,52^ and our results showed that MOR-triggered activation of PI3K/Akt further activated MAPK to promote the development of morphine tolerance. Blockade of spinal D2DR inhibited MOR-triggered activation of the PI3K/Akt-MAPK signaling pathway, which provided a new target for the prevention or reduction of morphine tolerance.

In conclusion, MOR-triggered activation of the PI3K/Akt-MAPK signaling pathway in the spinal cord is the mechanism underlying the development of morphine tolerance. Blockade of spinal D2DR attenuates morphine tolerance by inhibiting the activated downstream PI3K/Akt-MAPK signaling pathway in a MOR-dependent manner. *l-*CDL antagonizes spinal D2DR to inhibit the activation of the PI3K/Akt-MAPK signaling pathway in a MOR-dependent manner to attenuate morphine tolerance.

## Electronic supplementary material

Supplementary information
