# Supplementary material for: Selective blockade of spinal D2DR by levo-corydalmine attenuates morphine tolerance via suppressing PI3K/Akt-MAPK signaling in a MOR-dependent manner
Source: Exp Mol Med. 2018 Nov 14;50(11):148. doi: 10.1038/s12276-018-0175-1 (PMC6235923; doi:10.1038/s12276-018-0175-1)
Supplement: Supplementary file 1 — Supplementary information [file 12276_2018_175_MOESM1_ESM.doc]

**Selective blockade of spinal D2DR by *levo*-Corydalmine attenuates morphine tolerance via suppressing PI3K/Akt-MAPK signaling in a MOR dependent manner**

Wen-Ling Dai ^1^, Xin-Tong Liu ^2^, Yi-Ni Bao ^1^, Bing Yan ^1^, Nan Jiang ^1^, Bo-Yang Yu ^3*^, Ji-Hua Liu ^1, 3*^

**Supplemental Experimental Procedures**

*locomotor Activity Test*

The locomotor activity test was conducted with apparatus which consists of a round box surrounded by six infrared detectors. The mouse was put into the box to adapt for 1 min and then the locomotor activity was recorded by the detectors in 5 min. The locomotor activity was the accumulation of detected signals in 5 min.^1^ *l-*CDL (5, 10, 20mg/kg) at analgesic doses^2, 3^ was administered intragastrically to test its effect on locomotor activity of mice.

*Primary cultures neurons of spinal cord*

The spinal cords of the embryo mice were removed aseptically on day 13 of gestation.^4^ The primary neurons were isolated and cultured as described before.^5^ The spinal cords were digested in 0.15% trypsin at 37 °C for 25 min. The cell suspension was centrifuged at 1000 rpm for 4 min at room temperature. The sediment was resuspended and dissociated by slowly pipetting up-and-down 4-6 times with the smallest fire-polished glass Pasteur pipette. The cell suspension was sedimentated and centrifuged at 1000 rpm for 4 min. Neurobasal plating medium (Gibco, Gaithersburg, MD, USA) containing 10% FBS supplement and 1% *l-*glutamine were added. Cells were planted onto poly-*D-*lysine-pretreated 96-well (9 mm) plates with a density of 1.0 × 10^5^ cells/well. Cells were incubated at 37 °C with 5% CO_2_ and 95% humidity. Cytosine arabinoside (10 μM) was added to the culture medium on day 3 after planting to prevent proliferation of non-neuronal cells. The planting medium was replaced on day 5 with the neurobasal growth medium containing 10% B27, 1% *l-*glutamine and 1% HEPES.

*MTT assay*

Primary cultures neurons of spinal cord (1.0 × 10^5^/well) were seeded into a 96-well plate. The cells were treated with *l*-CDL (50μM, 30μM, 10μM, 3μM, 1μM, 0.3μM, 0.1μM) at 7 day in vitro for 24h. Then *l-*CDL was removed and 100μl of 5 mg/ml methyl-thiazolyl-tetrazolium (MTT) was added to each cell. And the plates were incubated at 37˚C for 4 h. 150 μL dimethyl sulfoxide was added to each well after removal of the old medium, and absorbance was measured at 570/650 nm using a multi-well spectrophotometer (Bio-Rad, Hercules, CA, USA).

**Supplemental Results**

**Figure s1**


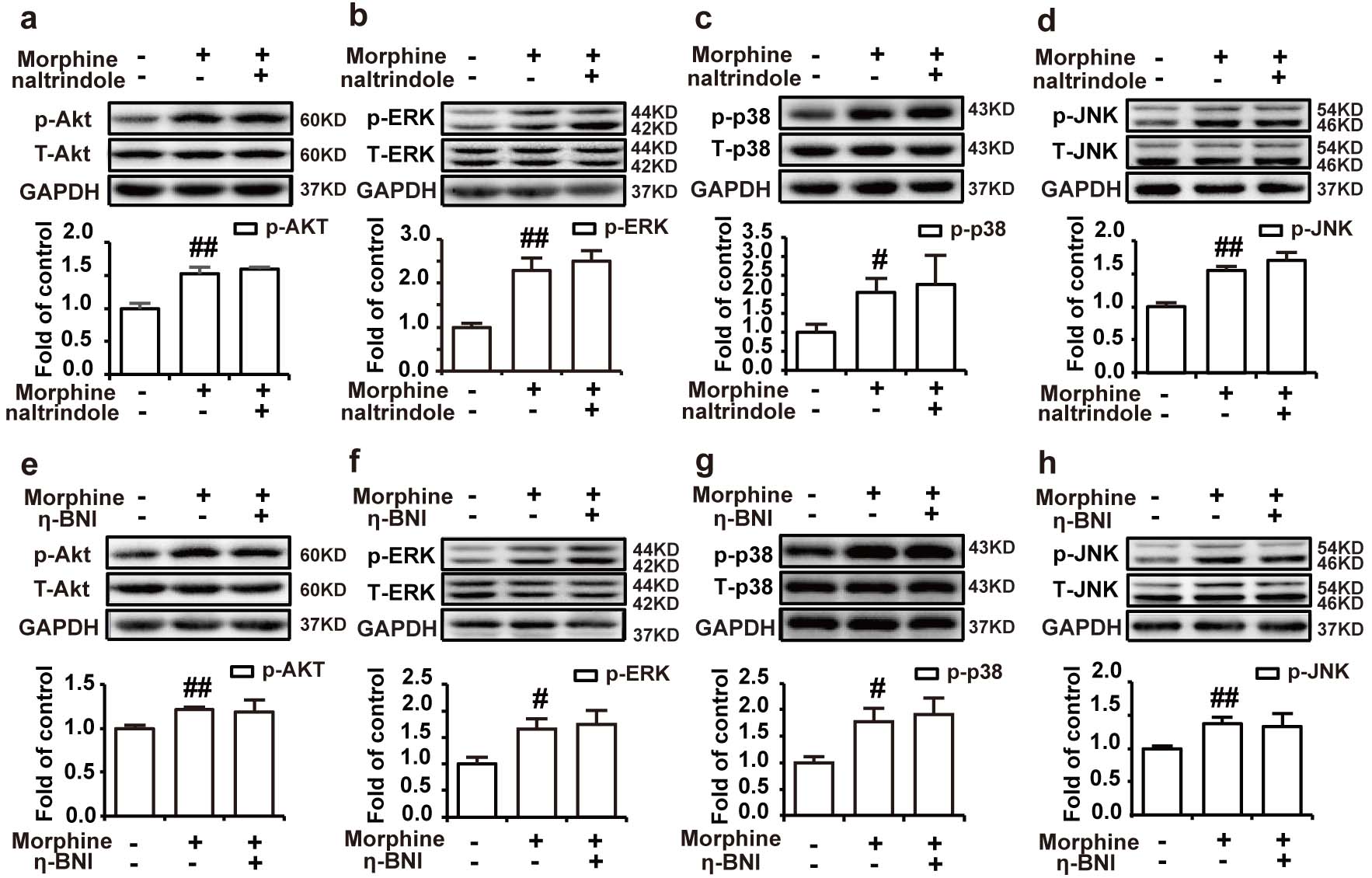


**Supplemental Figure s1** δ and κ opioid receptor are not involved in morphine tolerance induced PI3K/Akt and MAPK activation in the spinal cord. (a, b, c and d) Intrathecal administration of selective δ opioid receptor antagonist naltrindole (1 ng/10μl, i.t., 15 minutes before morphine treatment) could not reverse the increased expression of p-Akt, p-ERK1/2, p-p38 and p-JNK in the spinal cord. (e, f, g and h) Intrathecal administration of selective κ opioid receptor antagonist η-BNI (1 ng/10μl, i.t., 15 minutes before morphine treatment) also could not reduce the upregulated expression of p-Akt, p-ERK1/2, p-p38 and p-JNK in the spinal cord. Mice were examined daily with tail-flick assay. Data were shown as percentage of maximal possible effect (% MPE). Data presented as the mean ± SE. n = 4, #*P*＜0.05, ##*P*＜0.01, compared with control group.

**Figure s2**


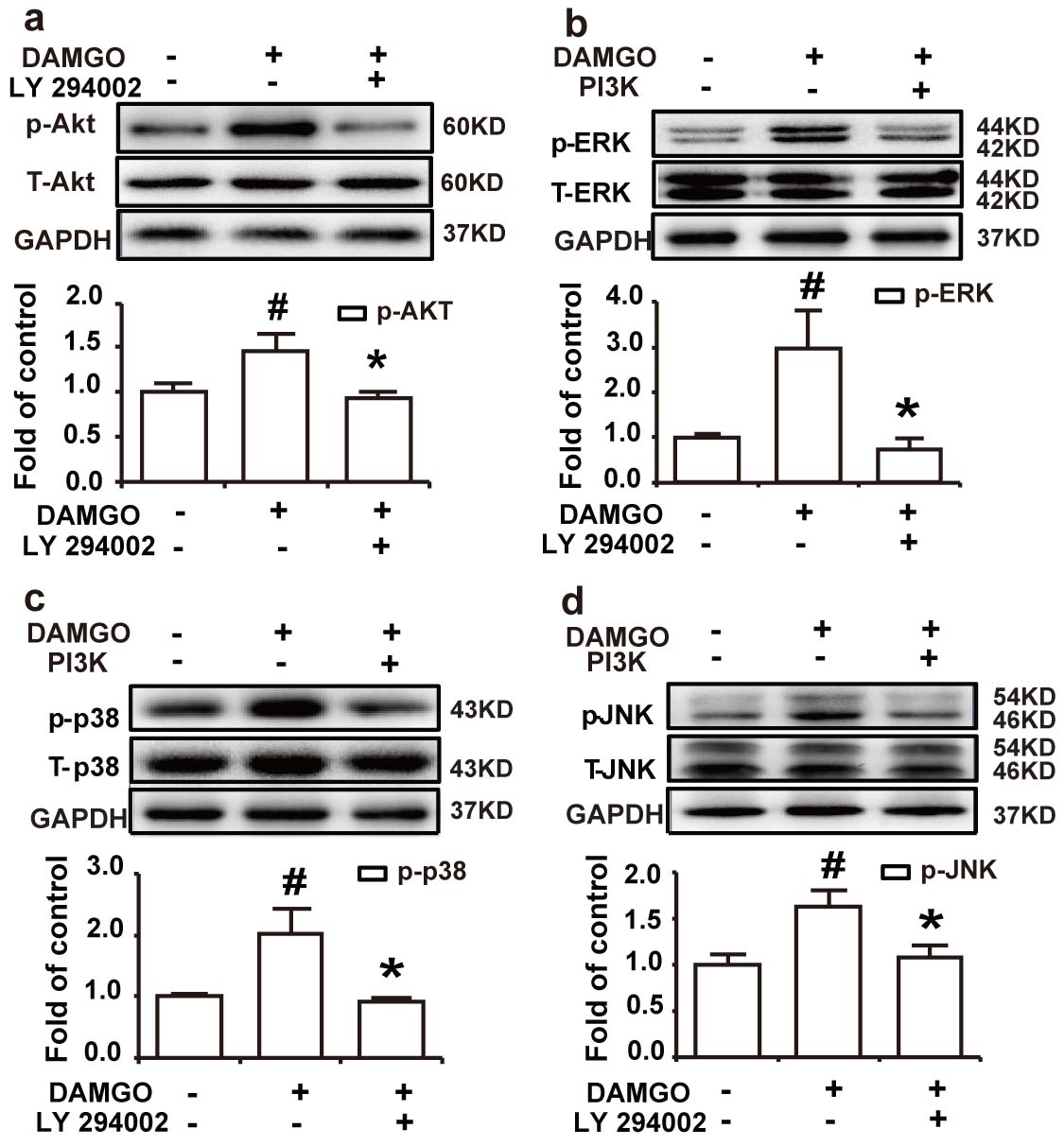


**Supplemental Figure s2** PI3K inhibitor could inhibit DAMGO induced upregulation of p-Akt and p-MAPK in the spinal cord. (a) Intrathecal administration of PI3K inhibitor could decrease DAMGO induced upregulation of p-Akt in the spinal cord. (b, c and d) Intrathecal administration of PI3K inhibitor could also decrease the upregulated expression of p-MAPKs in the spinal cord Data presented as the mean ± SE. n = 4, #*P*＜0.05, ##*P*＜0.01, compared with control; **P*＜0.05, ***P*＜0.01, compared with DAMGO group.

**Figure s3**


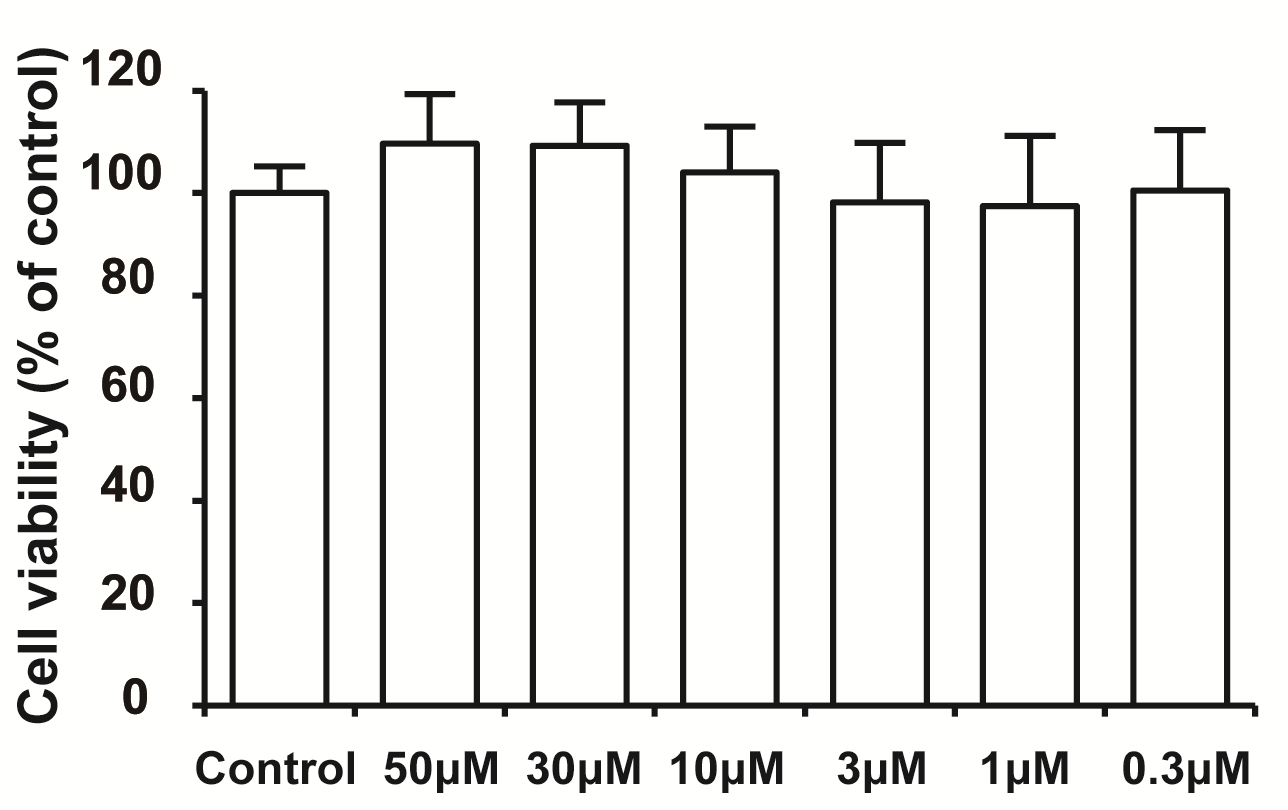


**Supplemental Figure s3** Cell survival was assessed by MTT assay. Administration of different doses of *l-*CDL (50, 30, 10, 3, 1, 0.3, 0.1μM) for 24 h has no effect on the viability of primary cultures neurons, n=5.

**
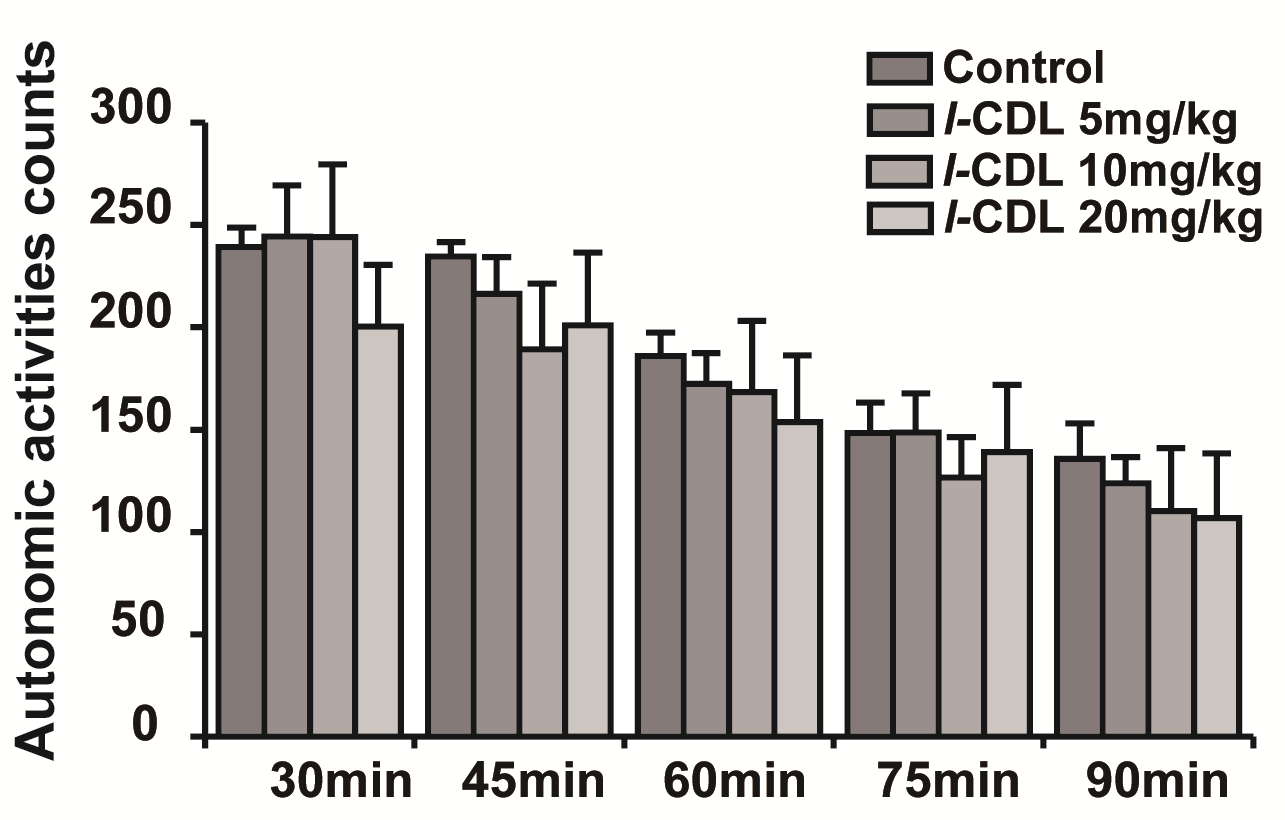
**

**Figure s4** There were no differences among groups in the locomotor activities of normal mice or mice intragastrically administrated *l-*CDL (5, 10, 20 mg/kg) at 30min, 45min, 60min, 75min and 90min, n=8.

**References:**

1. Lian W, Jia H, Xu L, Zhou W, Kang, Liu A et al. Multi-protection of DL0410 in ameliorating cognitive defects in D-galactose induced aging mice. *Front Aging Neurosci* 2017; **9**: 409.

2. Zhou L, Hu Y, Li C, Yan Y, Ao L, Yu B et al. Levo-corydalmine alleviates vincristine-induced neuropathic pain in mice by inhibiting an NF-kappa B-dependent CXCL1/CXCR2 signaling pathway. *Neuropharmacology* 2018; **135**: 34-47.

3. Hu Y, Kodithuwakku ND, Zhou L, Li C, Han D, Fang W et al. Levo-corydalmine alleviates neuropathic cancer pain induced by tumor compression via the CCL2/CCR2 Pathway. *Molecules* 2017; **22:** 937.

4. Langlois SD, Morin S, Yam PT, Charron F. Dissection and culture of commissural neurons from embryonic spinal cord*.* *J Vis Exp* 2010; **25**: e1773.

5. Dai WL, Yan B, Jiang N, Wu JJ, Liu XF, Liu JH et al. Simultaneous inhibition of NMDA and mGlu1/5 receptors by levo-corydalmine in rat spinal cord attenuates bone cancer pain. *Int J Cancer* 2017; **141**: 805-815.
